# Innovative Device and Procedure for In Situ Quantification of the Self-Healing Ability and Kinetics of Self-Healing of Polymeric Materials

**DOI:** 10.3390/polym15092152

**Published:** 2023-04-30

**Authors:** Yuliet Paez-Amieva, Jaime Carpena-Montesinos, José Miguel Martín-Martínez

**Affiliations:** Adhesion and Adhesives Laboratory, University of Alicante, 03080 Alicante, Spain; yuliet.paez@ua.es (Y.P.-A.); j.carpena@ua.es (J.C.-M.)

**Keywords:** self-healing, self-healing ability, kinetics of self-healing, in situ quantification, polymer, polyurethane

## Abstract

A new device and procedure for the in situ quantification of the extent of the self-healing and the kinetics of self-healing of polymeric materials were proposed. The device consisted of flowing an inert gas below the sample placed in a hermetically closed chamber. When the sample was perforated/damaged, the gas passed through the hole made in the polymeric material and the gas flow rate declined as the self-healing was produced. Once the gas flow rate stopped, the self-healing was completed. The proposed method was simple, quick, and reproducible, and several in situ self-healing experiments at different temperatures could be performed in the same sample. As a proof of concept, the new device and method have been used for measuring the self-healing ability of different polyurethanes.

## 1. Introduction

Inspired by living tissues and because of the potential in different strategic technological areas, there has been a recent interest in the development of self-healing materials [1]. Self-healing materials may enable the reconstruction of fractured into intact structures [2,3,4,5,6]. Although materials with different self-healing abilities have been developed already, one of the issues that remains unsolved, particularly in polymeric materials, is to find a method for monitoring the self-healing ability and the kinetics of self-healing in a quantitative, repetitive, and precise manner.

Several qualitative methods have been proposed in the existing literature for a rough estimation of the self-healing ability of different materials [7,8,9,10]. The majority of those methods provide the time of the self-healing of the material and the self-healing is monitored mainly by visual inspection. Optical microscopy has been used for the qualitative assignment of the self-healing of polymeric materials [11,12,13,14]. The operational procedure consists of scraping or cutting the surface of the polymer with a blade or scissors, respectively, followed by the joining of the cut pieces, and the monitoring of the evolution of the self-healing in the microscope [15,16,17]. Similarly, scanning electron microscopy (SEM) [18,19] and confocal laser microscopy [20] have been used for the qualitative assessment of the self-healing ability of polymers. The optical methods are not able to assess the extent of the self-healing or its kinetics. Furthermore, the edges of the cut are not flat, a scar is produced in the self-healed part, and the reproducibility of the method is poor. On the other hand, the self-healing cannot be assessed more than once in the cut zone, and it is not possible to distinguish whether the self-healing occurs from the surface to the bulk or from the bulk to the surface.

A less common method for the qualitative evaluation of the self-healing ability of polymers is the measurement of the conductivity [21]. In this method, the material must be coated with a highly conductive silver or galinstan (GaInSn) ink [15,22]. The conductivity of the as-received polymer is measured and then, a cut with a blade is made which removes the electrical conductivity. The two separated halves of the polymer are joined and once the self-healing occurs, all or part of the conductivity is recovered. Although this method is simple and relatively fast, the application of a conductive coating on the polymer is difficult to control due to its small thickness.

The simplest, fastest, and easiest method to assess the self-healing of a material is to make a cut with scissors and join the cut parts at a given temperature. After a given elapsed time, a mechanical test of the repaired material is carried out to determine the extent of the self-healing [23]. This method is indicative of the existence of self-healing in materials and allows for an approximate estimation of the self-healing time. In addition, this method does not allow for the measurement of the kinetics of self-healing.

Several quantitative methods for assessing the self-healing ability of materials have been developed and they relate the self-healing with a property of the material, such as its mechanical resistance to traction and/or rupture or its rheological properties, among others [24,25,26,27,28,29,30].

One of the few methods for the quantitative assessment of the self-healing of materials is the so-called “metallic wire” method. The method consists of passing a 1 mm in diameter metal wire, with a given weight hanging on the sides, through the center of a cylindrical or rectangular polymer piece. As the wire passes through the piece, the damaged area regains its structural integrity at room temperature [31]. Although it seems a simple method, it requires a complicated preparation, and the material needs an adequate geometry. In addition, the material must be rectangular or cylindrical with a significant thickness to enable a slow pass of the metal wire through the sample. If the material is very soft, the piece deforms and even breaks irreversibly, and the self-healing cannot be measured. Therefore, the method is restricted to materials with suitable mechanical properties.

The quantitative methods more widely used for assessing the self-healing ability of materials are mechanical (strain–stress) tests [16,32,33,34,35,36,37,38,39]. The mechanical properties are measured in dog-bone-shaped specimens that are later cut with a sharp knife into two equal parts. The cut pieces are carefully joined and allowed to heal at a given temperature for a given time. The mechanical properties of the joined dog-bone test specimen are tested in a universal testing machine at a constant pulling rate [20]. Both the force and the elongation are measured during the deformation of the sample until it breaks. With the data extracted from the mechanical test, the self-healing efficiency can be estimated by Equation (1) [16]:

Healing efficiency (%) = [(Tensile strength)/(Original tensile strength)] × 100(1)

In general, this method is very laborious and not very reproducible. In addition, after joining the cut pieces, the mechanical properties of the as-received material are usually not recovered.

Another method to quantify the self-healing of materials is the pull-off test [40,41]. This method consists of cutting the material with a razor blade into two equal parts followed by an alignment of the two halves in the jaws of a micro-testing machine [42]. After alignment, the two halves of the sample are compressed at a certain force for a given time, and a pull test is carried out; the measured force of the material is related to its degree of self-healing. This method is laborious and difficult to control. In addition, the pulling rate, the sample size, and the need for a precise alignment of the two cut pieces affect the extent of the self-healing. One of the advantages of this method is that it enables the evaluation of the self-healing in damaged samples under “non-ideal” conditions such as the misalignment of the specimens, the non-flat contacting surfaces, or in the cases in which an incomplete self-healing is produced.

A device and a method for determining the width of a crack during the self-healing of concrete materials were proposed in KR102105840 patent [43]. The method evaluates the self-healing of the concrete materials from the gas diffusion coefficients toward the crack. The method consists of making a crack in a concrete piece and placing it in contact with the upper part of a hermetic chamber filled with a pressurized gas. Then, the pressurized gas is allowed to flow out of the chamber into the crack, and the changes in the gas concentration over time are recorded to calculate the gas diffusion coefficient. The extent of the self-healing of the concrete material is estimated from the variation in the gas diffusion coefficients at different pressures. This method has several limitations: (i) the crack in the concrete material is not generated “in situ”, so the self-healing may begin before the measurement starts; (ii) the opening of the hermetic pressurized gas chamber must coincide exactly with the crack in the material to avoid gas leaks, which is not easy considering that the shape of the crack is irregular; (iii) the method is only valid for mainly elastic materials, so it is limited to flexible or soft materials (such as the most polymeric materials); and (iv) the repeatability is limited because the crack is made manually and outside of the device.

The revision of the current literature has shown that, until now, no method or device allows for an in situ quantitative measurement and monitoring of the self-healing process of polymeric materials. The proposed methods are labor intensive, they are not reproducible, the kinetics of self-healing are not measured, repetitive measurements at different temperatures in the same sample are not feasible, and the self-healing is related to the variation in one given property of the material. In this study, a new device for the in situ assessment of the self-healing of polymeric materials, as well as for monitoring their kinetics of self-healing, is proposed. In addition, a procedure for evaluating and monitoring the self-healing of polymeric materials is proposed; the procedure consists of perforating the polymer, allowing a flow of an inert gas to pass through the damaged zone. The decrease in the gas flow rate through the perforation made in the polymeric material is related to the kinetics of self-healing. When the gas flow rate is stopped, the self-healing is completed. This new method is simple, fast, and reproducible, and allows for several in situ measurements on the same sample at different temperatures. The new device and procedure have been tested in polyurethanes showing different self-healing abilities at room temperature, but there is potential for the use of the new device in other polymers, particularly for electronics [44].

## 2. Materials and Methods

### 2.1. Synthesis of the Polyurethanes

The polyurethanes were synthesized by using the one-shot method. The reactants used in the synthesis of the polyurethanes were 4,4′-methylene bis (cyclohexyl isocyanate) (HMDI) with 90% purity (Sigma Aldrich Co., St. Louis, MO, USA), 1,4 butanediol (BD) with 99% purity (Panreac Applichem^®^, Darmstadt, Germany), and polycarbonate of 1.6 hexanediol polyols with different molecular weights—Eternacoll^®^ UH-100 (UBE Chemical Europe S.A., Castellón, Spain) with molecular weight of 1000 Da, and Eternacoll^®^ UH-200 (UBE Chemical Europe S.A., Castellón, Spain) with molecular weight of 2000 Da. An NCO/OH ratio of 1.1 was used.

All polyurethanes were synthesized by using the same procedure and they differed only in the molecular weight of the polyol or in the composition of the polyols blend:

—100% YPD polyurethane—synthesized with the polycarbonate of 1.6 hexanediol polyol with molecular weight of 1000 Da.
—100% YPD2 polyurethane—synthesized with the polycarbonate of 1.6 hexanediol polyol with molecular weight of 2000 Da.
—60%YPD40%YPD2 polyurethane—synthesized with a blend of 60 wt.% polycarbonate of 1.6 hexanediol polyol with molecular weight of 1000 Da and 40 wt.% polycarbonate of 1.6 hexanediol polyol with molecular weight of 2000 Da.
—20%YPD80%YPD2 polyurethane—synthesized with a blend of 20 wt.% polycarbonate of 1.6 hexanediol polyol with molecular weight of 1000 Da and 80 wt.% polycarbonate of 1.6 hexanediol polyol with molecular weight of 2000 Da.

To synthesize the polyurethanes, the required amount of polyol/polyols blend and 1,4-butanediol was added to a 60 mL polypropylene bottle, the mixture was stirred in a SpeedMixer DAC 150.1 FVZ-K device (FlackTek Inc., Landrum, SC, USA) at 2400 rpm for 1 min. Then, the mixture was placed in an oven at 80 °C for 10 min. Subsequently, the required amount of HMDI (isocyanate) was added to the mixture and stirred again in a SpeedMixer device at 2400 rpm for 1 min. Finally, the mixture was placed in a vacuum oven for 8 h and heated by following different consecutive stages: 50 °C for 30 min; 60 °C for 30 min; 70 °C for 30 min; and 80 °C for 6 h and 30 min. After 24 h at room temperature, an annealing process was carried out at 85 °C for 1 h to stabilize the structure of the polyurethanes.

### 2.2. Device for In Situ Quantification and Monitoring of the Self-Healing of Polymeric Materials

**Figure 1:**
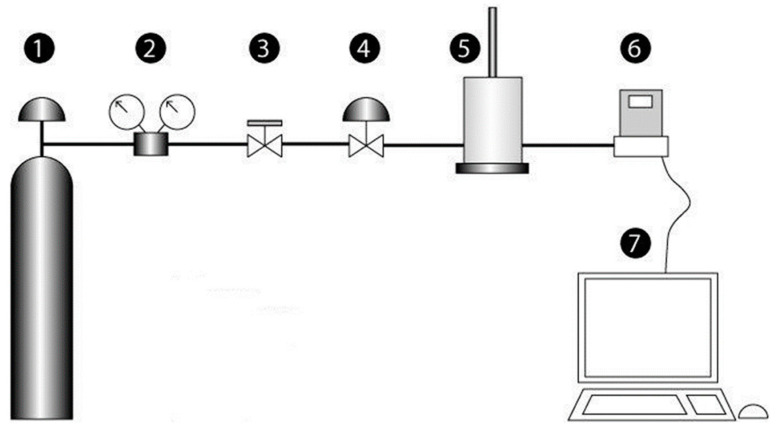
Scheme of the new device for measuring the self-healing of polymeric materials. 1—inert gas container; 2—gas pressure regulator; 3—shut-off valve; 4—gas flow control valve; 5—chamber containing the sample; 6—gas flow meter; 7—data acquisition device.

The device consists of an inert gas container, a pressure regulator, a shut-off valve, a gas flow control valve (Swagelok, Solon, OH, USA), a chamber containing the sample, a flow meter, and a data acquisition device (Figure 1). One key requirement of the device is the accurate control of the gas flow rate as its decrease with time is related to the extent of self-healing of the sample. Thus, parts 1 to 4 of the device in Figure 1 are intended for accurate gas flow control and part 6 is an accurate electronic gas flow controller (FG-111B, Ruurlo, The Netherlands).

In the new device, the risk of undesirable potential chemical reactions of the gas with the polyurethane during the self-healing process was avoided. Therefore, reactive gases such as oxygen or carbon dioxide were not tested. Dried inert gases (nitrogen, argon, helium) were used. The self-healing ability of the polyurethanes determined by using dried helium, argon, and nitrogen gases were similar and, due to its lower cost, dried nitrogen gas (99.9998% purity, Linde, Barcelona, Spain) was chosen.

**Figure 2:**
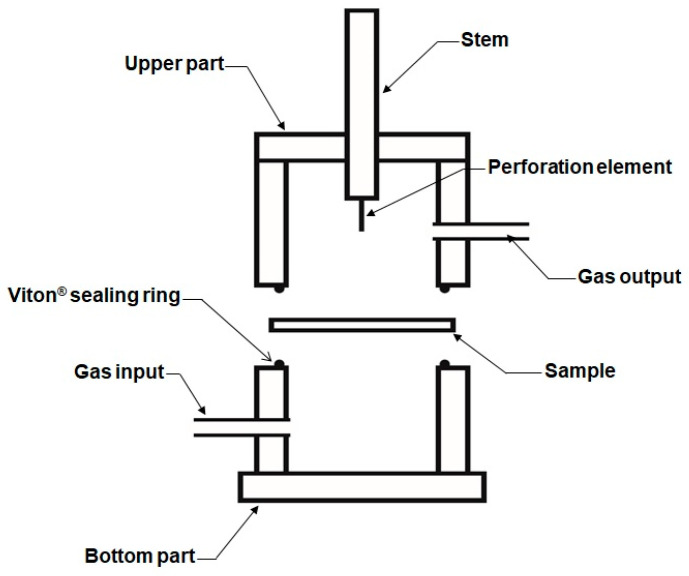
Diagram of the chamber containing the sample in which the self-healing is measured.

Figure 2 shows the detailed description of the chamber containing the sample (part 5 of Figure 1) in which the self-healing is measured. The cylindrical chamber with a diameter of 50 mm and a height of 76 mm is made of Teflon^®^, and consists of two parts that are joined by screws. The sample is placed in the middle and tightened when closed by means of Viton^®^ sealing rings located in the perimeter of the upper and bottom parts of the chamber. This design assures a hermetic cavity for avoiding gas leaks during the measurement. The chamber has a gas input and a gas output entry located below and above the sample, respectively. A stem tipped with a stainless steel needle of 0.5–1.25 mm in diameter is placed in the center of the upper part of the chamber; the stem may slide vertically up and down (Figure 2).

On the other hand, the chamber is surrounded by an electric wire coupled to a thermocouple placed in the upper part of the chamber and in the proximity of the sample surface. This electric wire permits the heating of the chamber up to 150 °C. The chamber is covered by a heat insulator (glass wool) to avoid heat losses. For simplicity, Figure 2 and their different parts are not at scale and the heating element is not shown.

**Figure 3:**
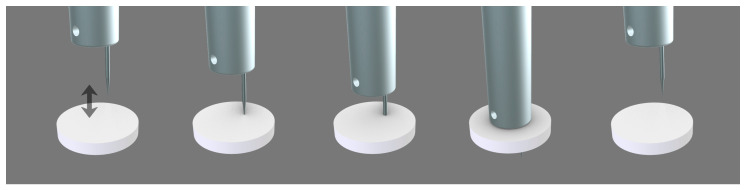
Movement of the stem for perforating the polymeric material in the chamber.

**Figure 4:**
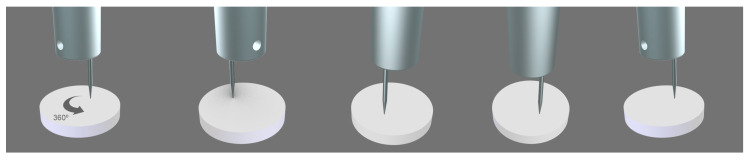
Eccentric drilling of the stem for perforating the polymeric material in different locations.

When the two parts of the chamber of the device with the sample in the middle are closed, the stem slides down vertically to allow the needle to pierce and transverse the sample in full. Then, the stem slides up to allow the gas to flow from bottom upward through the punctured zone of the sample (Figure 3). The stem and the perforating element may rotate 360° to allow for the drilling of the same sample at any point. In fact, the needle is not placed in the center of the stem, but on one side to enable different repetitions of the self-healing in the same sample (Figure 4).

### 2.3. Procedure for In Situ Measurement of the Self-Healing of Polymeric Materials

**Figure 5:**
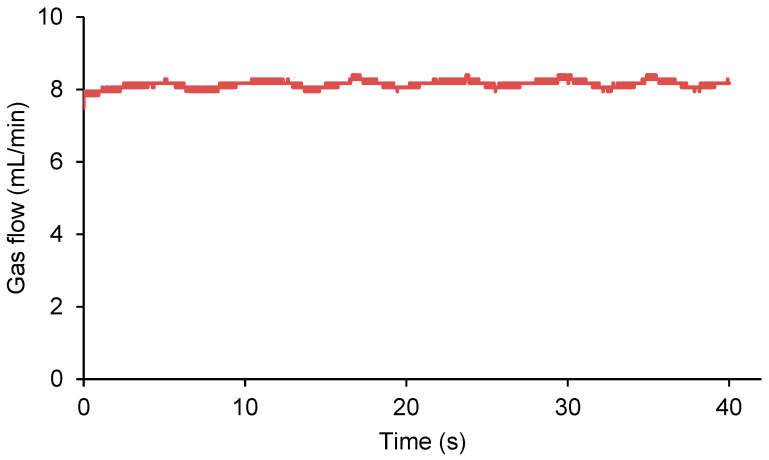
Variation in the gas flow as a function of time in the chamber of the measuring device prior to puncturing the sample.

The procedure for quantifying and monitoring the self-healing of polymeric materials needs a hermetic sealing of the sample between the two parts of the chamber. To ensure that the pressure in the chamber of the measuring device is uniform prior to puncturing the polyurethane specimens, the gas flow rate was monitored over time. A gas flow of 8 mL/min was selected and the variation in the gas flow rate over time was monitored. According to Figure 5, the gas flow is maintained constant over 40 s, a time higher than the one needed for the self-healing of the polyurethanes used in this study.

**Figure 6:**
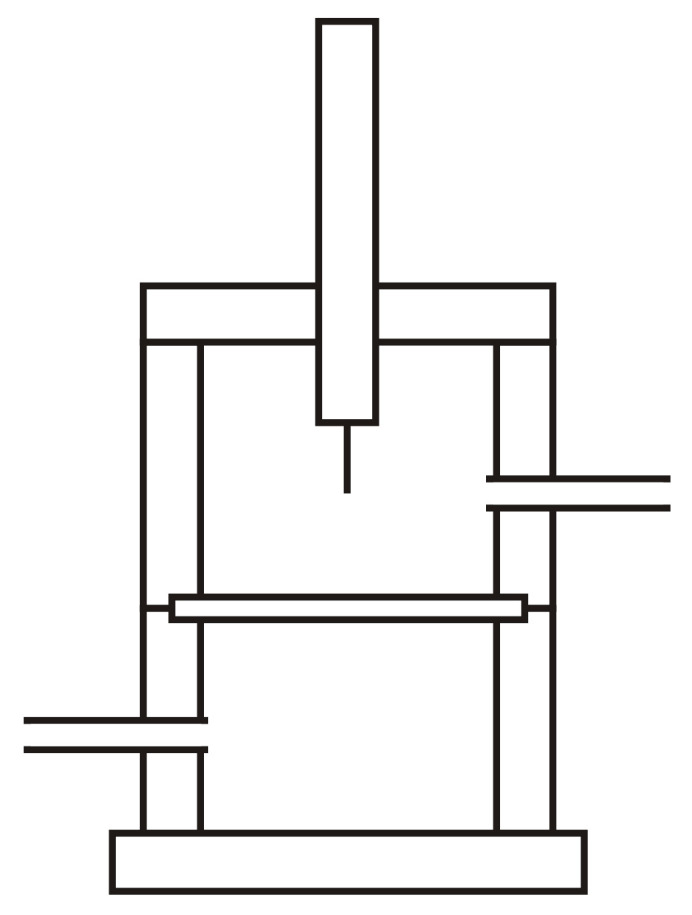
Sample of polymeric material placed in the center of the chamber.

A circular-shaped polymeric material sample with a diameter of 19 mm and a thickness of 3 mm is placed in between the two parts of the chamber. When the chamber is closed, the polymer sample is tightened by means of Viton^®^ sealing rings located in the perimeter of the upper and bottom parts (Figure 6). The diameter of the sample is lower than the section of the chamber which has a diameter of 50 mm.

**Figure 7:**
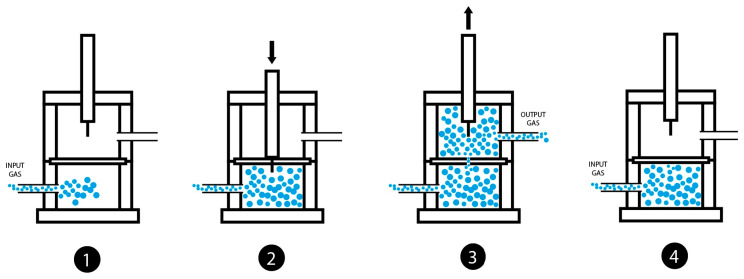
Diagram of the procedure for measuring the self-healing of polymeric materials in the chamber. 1—gas inlet at the bottom of the chamber; 2—puncture of the sample; 3—removal of the stem to allow the gas to flow through the damaged zone of the sample; 4—cloture of the damaged zone of the sample (the gas flow is stopped).

Figure 7 shows the steps followed in the monitoring of the self-healing of a polymeric material placed in the chamber. During the duration of the measurement, a constant continuous small flow of an inert gas (8–65 mL/min) flows to the bottom part of the chamber. The gas is allowed to flow just before the sample is placed in the chamber (step 1 in Figure 7) and immediately afterward, the stem oriented by the shank approaches the sample to be eccentrically drilled with the needle (step 2 in Figure 7). Then, the stem with the perforation device is removed from the sample and the gas starts to flow from bottom upward through the damaged zone of the sample (step 3 in Figure 7). The gas flow rate is monitored continuously over time until the gas flow is stopped, and, at this point, the self-healing is completed (step 4 in Figure 7).

## 3. Results

### 3.1. Qualitative Assessment of the Self-Healing of Polyurethanes

The self-healing of the 100% YPD and 100% YPD2 polyurethanes was assessed by cutting them with scissors. Videos S1 and S2 (Supplementary Materials file) show the cutting of the polyurethane samples with scissors, the joining of the cut parts at room temperature under moderate pressure for 15 s, and the manual tensile test of the joined parts. Video S1 (Supplementary Materials file) shows a complete self-healing in the 100% YPD polyurethane, whereas Video S2 (Supplementary Materials file) shows the absence of self-healing in the 100% YPD2 polyurethane.

Because the assessment of the self-healing of the polyurethanes by using scissors was quite rough, a new device for monitoring the self-healing ability of the polyurethanes at room temperature was developed in this study.

### 3.2. Monitoring of the Self-Healing of Polyurethanes by Using the New Device

**Figure 8:**
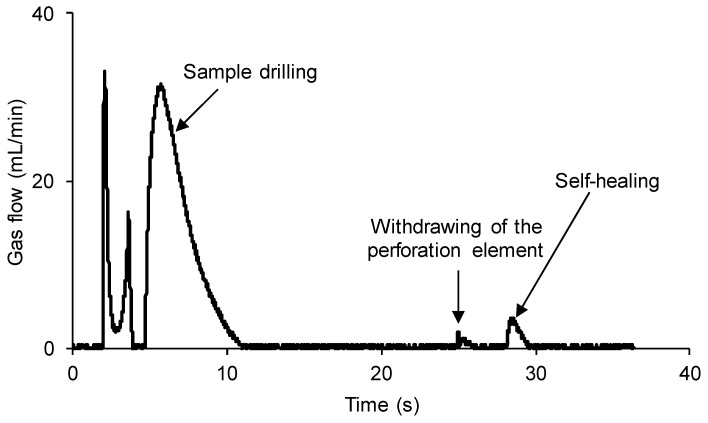
Variation in gas flow as a function of time for 100% YPD polyurethane showing the different stages of the self-healing measuring procedure.

A continuous flow of dried nitrogen gas at a pressure of 750 mbar and a flow rate of 8 mL/min was used. The 100% YPD polyurethane piece was completely drilled at 0.5 mm off the center of the sample with a needle of 1 mm in diameter attached to the stem. Figure 8 shows the variation in the gas flow with time at different stages of the self-healing assessment of the 100% YPD polyurethane. Before puncture, the gas was confined in the bottom part of the chamber, so an increase in pressure was reached. Figure 8 shows three differentiated processes: (i) the puncturing of the 100% YPD polyurethane generates a sudden increase in the gas flow (overpressure) which is confined in the bottom part of the chamber, followed by a sudden decrease in the gas flow rate until it becomes constant; (ii) the removal of the needle from the 100% YPD polyurethane generates a slight overpressure due to the gas stored in the bottom part of the chamber while the needle was placed in the sample; and (iii) the self-healing process in which the gas flow rate is continuously decreasing over time.

**Figure 9:**
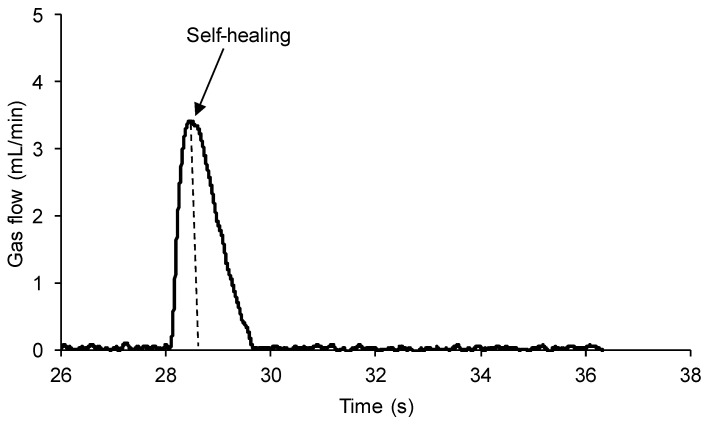
Variation in gas flow as a function of time for 100% YPD polyurethane in the self-healing region.

Figure 9 shows the region where the self-healing of the 100% YPD polyurethane occurs. The self-healing of the 100% YPD polyurethane is produced quickly and is finished in 1.4 s.

**Figure 10:**
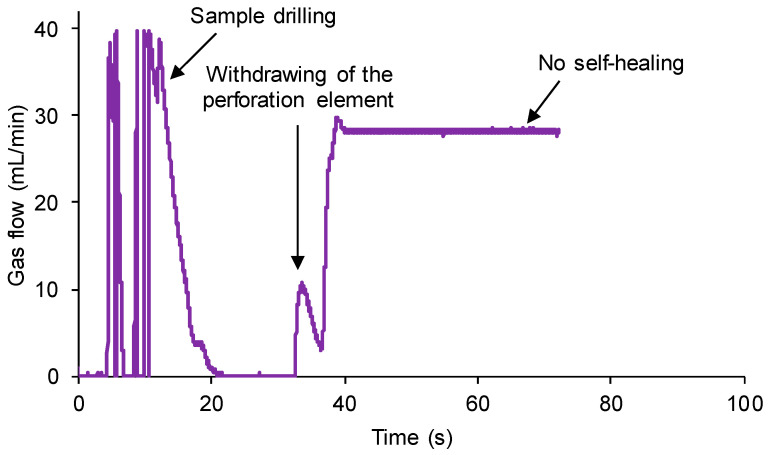
Variation in gas flow as a function of time for 100% YPD2 polyurethane showing the different stages of the self-healing measuring procedure.

The same experiment was repeated for a polyurethane without self-healing ability (100% YPD2). A continuous flow of nitrogen gas at a pressure of 750 mbar and a flow rate of 8 mL/min was produced. The 100% YPD2 polyurethane piece was completely drilled at 0.5 mm off the center of the sample by using a needle of 1 mm in diameter attached to the stem. Figure 10 shows the variation in the gas flow with time at different stages of the self-healing assessment of the 100% YPD2 polyurethane. Three differentiated processes are distinguished: (i) the puncture of the 100% YPD2 polyurethane generates a sudden increase in the gas flow (overpressure) which is confined in the bottom part of the chamber, followed by a sudden decrease in the gas flow rate until it becomes constant; (ii) the removal of the needle from the 100% YPD2 polyurethane generates a slight overpressure due to the gas stored at the bottom part of the chamber while the needle was placed in the sample; and (iii) the gas flow is high and constant over time indicating that the self-healing of the 100% YPD2 polyurethane does not occur. Therefore, the new device and procedure are sensitive to assess the self-healing of the polyurethanes.

**Figure 11:**
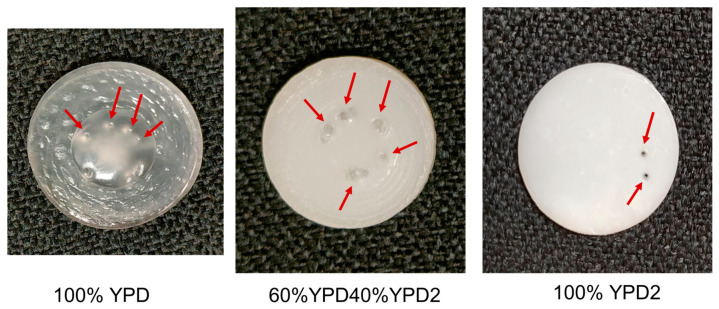
Photos of the polyurethanes after self-healing assessment experiments. The red arrows show the location of the punctures made during the experiments.

After carrying out the experiments for the assessment of the self-healing of the polyurethanes, the tested samples were monitored by visual inspection. Figure 11 shows the photos of three polyurethanes after the self-healing assessment experiments. The several punctures made in the polyurethanes can be noticed (they are marked with red arrows), and all of them appear fully closed in the 100% YPD and 60%YPD40%YPD2 polyurethanes and fully open in the 100% YPD2 polyurethane. Therefore, the experimental set-up proposed in this study is able to assess the self-healing of the polyurethanes.

### 3.3. Monitoring the Kinetics of Self-Healing and the Self-Healing Time of the Polyurethanes

Three polyurethanes with different self-healing abilities and one polyurethane which does not show self-healing have been selected in this study for testing the performance of the new device in monitoring the kinetics of self-healing and the self-healing time.

**Figure 12:**
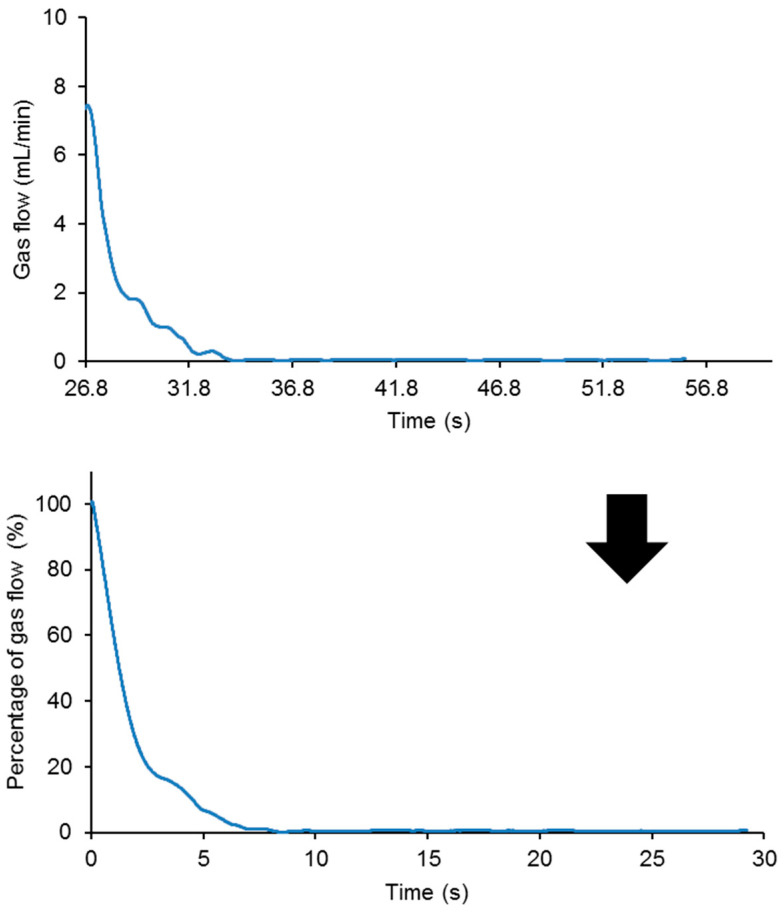
Variation in the gas flow (up) and the percentage of the gas flow (bottom) as a function of the time for 20%YPD80%YPD2 polyurethane.

A continuous flow of nitrogen gas at a pressure of 750 mbar and a flow rate of 8 mL/min was produced. The polyurethane pieces were completely drilled at 0.5 mm off the center by using a needle of 1 mm in diameter attached to the stem. For monitoring the kinetics of self-healing of the polyurethanes, the following procedure was used. The self-healing part of the plots of the variation in the gas flow as a function of the time of the polyurethanes was selected and the time was adjusted to zero at the beginning of the self-healing (Figure 12). Then, the gas flow rate at the beginning of the self-healing was fit to a percentage of 100%, and the decline in the gas flow rate over time was expressed as a percentage (Figure 12). Thus, the kinetics of self-healing of the polyurethanes are expressed as the percentage of the gas flow as a function of the time.

**Figure 13:**
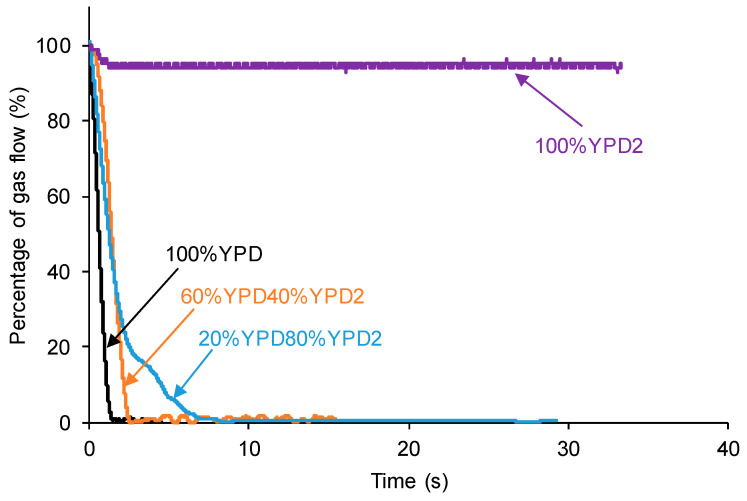
Kinetics of self-healing of different polyurethanes.

Figure 13 shows the variation in the percentage of the gas flow as a function of the time for the different polyurethanes. The self-healing of the 100% YPD and 60%YPD40%YPD2 polyurethanes is continuous and homogeneous, and is completed in 1.4 s and 2.5 s, respectively (Table 1). However, the self-healing of the 20%YPD80%YPD2 polyurethane is slower (it is completed in 8.5 s, Table 1) and occurs in several stages, being faster at the beginning and slowing down for times greater than 3 s. Finally, the 100% YPD2 polyurethane does not show self-healing. Therefore, the device and procedure allow for distinguishing between the different extents and kinetics of the self-healing in the polyurethanes.

### 3.4. Monitoring of the Self-Healing of the Polyurethanes by Using Different Gas Flow Rates

The gas flow rate may affect the self-healing of the polyurethanes. Therefore, the self-healing of the 100% YPD polyurethane (taken as a typical example) was monitored under the same experimental conditions, but by changing the nitrogen gas flow rate between 8 and 65 mL/min.

**Figure 14:**
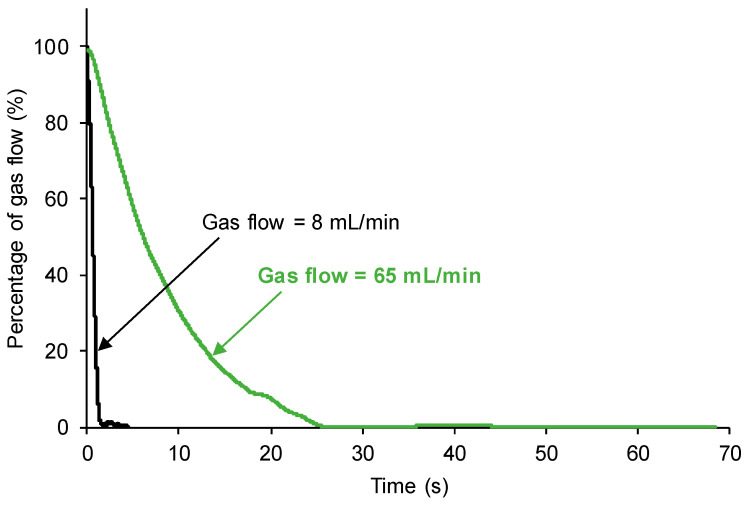
Kinetics of self-healing of 100% YPD polyurethane measured with different nitrogen gas flow rates.

Figure 14 shows that the increase in the gas flow rate increases the self-healing time of the 100% YPD polyurethane from 1.4 to 26 s (Table 2). In addition, when the nitrogen gas flow rate is small, the self-healing of the 100% YPD polyurethane is continuous and homogeneous, while when the flow rate is higher, the self-healing is gradual and slows down over 18 s. Therefore, the kinetics of self-healing and the self-healing time of the polyurethanes are affected by the gas flow rate; the higher the gas flow rate is, the longer the self-healing is.

### 3.5. Monitoring of the Self-Healing of the Polyurethanes by Using Needles of Different Diameters

Different needles of 0.5 to 5 mm in diameter for puncturing the polyurethanes were tested in the new device. When a needle with a diameter larger than 1.25 mm was used, the hole/puncture in the sample cannot be completely closed because the pressure caused by the continuous flow of the gas from the bottom of the sample impeded its complete self-healing. Therefore, several needles of 1.25 mm or lower in diameter were selected.

**Figure 15:**
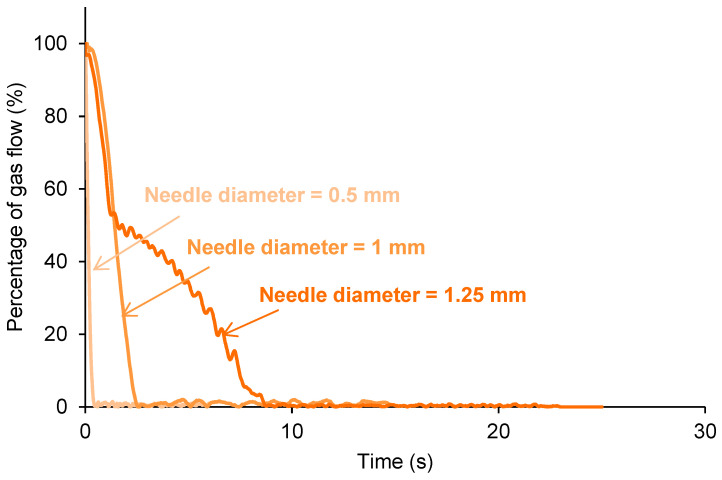
Kinetics of self-healing of 60%YPD40%YPD2 polyurethane monitored with needles of different diameters.

Figure 15 shows that the increase in the needle diameter increases the time required for the self-healing of the 60%YPD40%YPD2 polyurethane (taken as a typical example) from 0.4 to 8.6 s (Table 3). The increase in the self-healing time of the polyurethane is expected because the size of the hole is larger and it will take longer to be closed. In addition, when the needle diameter is small (i.e., 0.5 mm), the self-healing of the 60%YPD40%YPD2 polyurethane is continuous and homogeneous, while when the needle diameter is increased to 1.25 mm, the self-healing is gradual and slows down over 2.5 s. Therefore, the kinetics of self-healing and the self-healing times of the polyurethanes are affected by the needle diameter; the larger the needle diameter is, the longer the self-healing time is.

### 3.6. Reproducibility of the Self-Healing Assessment of the Polyurethanes

One important limitation of the procedures described in the existing literature for determining the self-healing of materials is the absence of reproducibility. The reproducibility of the procedure for assessing the self-healing proposed in this study was tested by repeating the self-healing measurement three times in three different locations of the same polyurethane. The nitrogen gas flow rate was set to 65 mL/min and the stem with a needle of 1 mm in diameter was rotated (Figure 4) and, in each position, the polyurethane was completely drilled.

**Figure 16:**
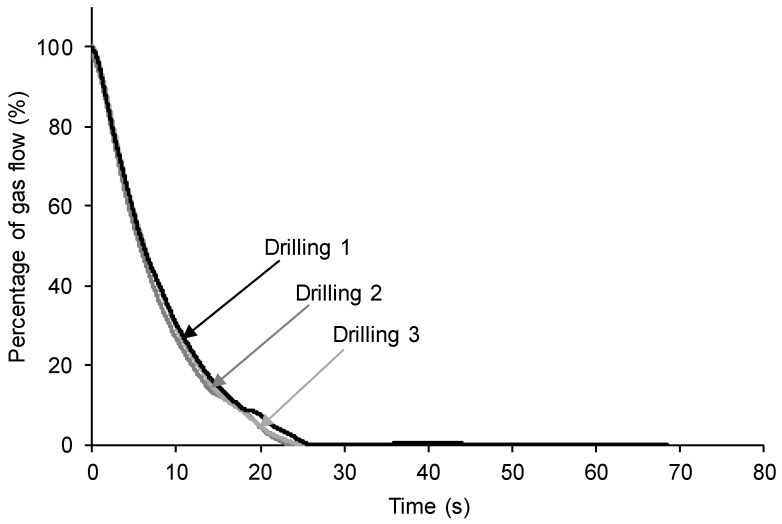
Kinetics of self-healing of 100% YPD polyurethane obtained by in situ drilling in three different zones.

Figure 16 shows that the self-healing times of the 100% YPD polyurethane, taken as a representative example, in the three measurements are very similar (24–26 s), and a similar kinetics of self-healing are obtained in the three repetitions (Table 4).

## 4. Discussion

In summary, the new device and procedure proposed in this study allows for quantifying the self-healing of different polyurethanes (Figure 8 and Figure 10). In addition, the distinct self-healing ability of the polyurethanes and their different kinetics of self-healing can be determined in situ (Figure 13). On the other hand, the device has several parameters that can be varied according to the conditions required for each polymer. Thus, if the polymer has a fast self-healing and a too short self-healing time (such as for the 100% YPD polyurethane), the gas flow rate (Figure 14) and/or the needle diameter can be increased (Figure 15) to increase the self-healing time. Furthermore, the increase in the gas flow rate allows for better monitoring of the kinetics of self-healing of the polyurethanes due to a fast self-healing ability. Finally, the reproducibility of the proposed device and procedure for determining the self-healing ability of the polyurethanes was demonstrated (Figure 16). This particular feature is extremely interesting because it opens up the possibility for the in situ testing of the self-healing of polymers in several locations and, if needed, at different temperatures.

## 5. Conclusions

In this work, a new device and procedure for the in situ quantification of the extent of the self-healing of polymers were proposed. The method was simple, fast, and reproducible. The experimental set-up allowed for the monitoring of the kinetics of self-healing. The device and procedure have been tested in polyurethanes with different self-healing abilities at room temperature. The new device allowed for different parameters to be varied depending on the specific characteristics of each polymer. The gas flow rate was varied between 8 and 65 mL/min and the needle diameter was changed between 0.5 and 1.25 mm. The increase in the gas flow rate and the needle diameter increased the duration of the self-healing process of the polyurethanes. Furthermore, the proposed method is reproducible and several in situ measurements of the extent of the self-healing can be made in the same sample.

## 6. Patents

A Spanish patent has been filed as a result of the work reported in this manuscript—patent filed on 16 February 2023. Application number: P202330118

## Figures and Tables

**Table 1:** Self-healing times of different polyurethanes.

PU	Self-Healing Time (s)
100%YPD	1.4
60%YPD40%YPD2	2.5
20%YPD80%YPD2	8.5
100%YPD2	No self-healing

**Table 2:** Self-healing times of 100% YPD polyurethane measured with different nitrogen gas flow rates.

Gas Flow Rate (mL/min)	Self-Healing Time (s)
8	1.4
65	26

**Table 3:** Self-healing times of 60%YPD40%YPD2 polyurethane monitored with needles of different diameters.

Needle Diameter (mm)	Self-Healing Time (s)
0.5	0.4
1 1.5	2.5 8.6

**Table 4:** Self-healing times of 100% YPD polyurethane obtained by in situ drilling in three different zones.

Drilling	Self-Healing Time (s)
1	26
2	25
3	24
Average	25 ± 1

## Data Availability

Not applicable.

## Supplementary Materials

The following supporting information can be downloaded at: https://www.mdpi.com/article/10.3390/polym15092152/s1, Video S1: Qualitative evaluation of the self-healing of the 100% YPD polyurethane monitored by cutting with scissors. Video S2: Absence of the self-healing of the 100% YPD2 polyurethane monitored by cutting with scissors.
